# Atrial fibrillation in Cardiac Channelopathies

**Published:** 2009-11-01

**Authors:** Jayachandran Thejus, Johnson Francis

**Affiliations:** Calicut Medical College, Kerala, India

**Keywords:** atrial fibrillation, cardiac channelopathies, Brugada syndrome, long QT syndrome, short QT syndrome, familial atrial fibrillation, common atrial fibrillation

## Introduction

Atrial fibrillation is the commonest arrhythmia encountered in day-to-day clinical practice. Its prevalence is increasing due to increasing age of the population, increasing prevalence of chronic heart disease and better diagnostic techniques. It is an extremely costly public health problem. It increases stroke, heart failure and mortality. Mortality is doubled by the presence of atrial fibrillation [1]. Extensive research is going on to understand better the genetic and molecular mechanisms underlying atrial fibrillation.

Cardiac channelopathies are diseases caused by mutations in genes encoding ion channels of the heart. Many cardiac channelopathies have been described. Atrial fibrillation is a manifestation of many of these channelopathies. The presence of atrial fibrillation often worsens the prognosis of these channelopathies and poses special problems in their diagnosis and management. Also, often atrial fibrillation is the presenting feature of a cardiac channelopathy- the other more ominous features appear only later. The management of atrial fibrillation is often different when it is due to a channelopathy. Thus understanding the relation between atrial fibrillation and the various cardiac channelopathies is very important.

## Brugada syndrome and atrial fibrillation

The commonest atrial arrhythmia in Brugada syndrome is atrial fibrillation. The incidence of AF in Brugada syndrome has been reported differently by different investigators and is in the range of 10% to 53% (Table 1) [2-6].

Three types of ECG patterns in right precordial leads are seen in Brugada syndrome. In type 1, coved ST-segment elevation ≥  2 mm is followed by a negative T wave. In type 2, ST elevation is saddleback with a takeoff ST elevation ≥  2 mm and a trough ≥  1 mm and the T wave is positive or biphasic. In type 3, there is saddleback or coved appearance and ST elevation is <  1 mm. Among the three ECG types of Brugada syndrome, atrial fibrillation is most likely to occur in type I. Kusano [4] found that in type 1, the incidence of spontaneous AF was 26.1% while in type 2 and 3 taken together, the incidence was only 8%. Bigi [6] found that AF occurred in 53% of type 1 patients while it did not occur in any type 2 or 3 patient.

Atrial fibrillation in Brugada syndrome is more likely (70%) to occur in the night [4]. In Brugada syndrome, ventricular fibrillation is also more likely to occur in the night [4]. Thus in Brugada syndrome, both atrial and ventricular fibrillation show a circadian rhythm. This indicates increased arrhythmogenecity at night in Brugada syndrome in both ventricles and atria, probably due to autonomic changes during sleep, that is, increased parasympathetic and decreased sympathetic activity. Though Brugada syndrome is more common in males, clear cut sex predilection has not been found for AF occurring in the setting of Brugada syndrome.

Various investigators have attempted to find predictors of development of atrial fibrillation in Brugada syndrome (Table 2). Previous life threatening cardiac events were found to predict AF in one study [6]. In another study, various ECG and EP parameters were found to predict AF; the most powerful predictor was HV interval more than or equal to 56 ms [8]. In another study, interatrial conduction delay was found to be significantly increased in Brugada syndrome patients with AF [4]. Notably, age is not a predictor of AF in Brugada syndrome [4,6] nor is the left atrial size [6]. Patients with spontaneous Brugada type ECG are more likely to have atrial arrhythmias when compared to those with inducible Brugada type ECG [9]. Chance of atrial arrhythmias is more in Brugada syndrome patients with ICDs implanted when compared with those without, probably due to more severe form of the disease and due to presence of more risk factors for sudden cardiac death [9,10]. Thus, the disease process in Brugada syndrome may be more advanced in those with AF than in those without. The studies by Kusano et al [4] and Bigi et al [6] have clearly shown that Brugada syndrome patients with AF are more likely to have syncope and ventricular fibrillation compared to those without AF. Letsas [8] found that increased P wave dispersion correlates with AF in Brugada syndrome while Bigi [6] found that it does not. 

Brugada syndrome is associated with known mutations in sodium and calcium channels in a minority of cases while in the majority, no mutation has been identified. In Brugada syndrome, atrial fibrillation can occur in all these mutations without predisposition to any particular mutation [4,9]. Atrial fibrillation is known to occur in SCN5A mutations unrelated to Brugada syndrome [11]. Such mutations are not documented in cases of atrial fibrillation occurring in the setting of Brugada syndrome. Genetic analysis may not be useful for risk stratification in Brugada syndrome as presence of SCN5A mutations in Brugada syndrome do not predict higher risk of VF [12] or AF [4] when compared to Brugada syndrome patients without these mutations.

The basis for ventricular arrhythmias in Brugada syndrome is an increase in I_to_, the transient outward current. Ito is prominent in the atria and hence the mechanism of AF in Brugada syndrome may be an increase in Ito in the atria [9]. AF in Brugada syndrome may be triggered by APCs [9]. Morita et al [5] have found that atrial vulnerability is increased in AF and have postulated that abnormal atrial conduction may be the electrophysiological basis for AF in Brugada syndrome.

In Brugada syndrome patients with ICD implanted, ICD shocks were found to be more commonly inappropriate than appropriate [2,13] in previous studies. A very recent study in 2009 by Veltmann et al [14] has found that appropriate shocks are slightly more common than inappropriate, probably reflecting better ICD programming. In this study, in Brugada syndrome patients who received ICDs, 2.91% per year received appropriate shocks while 2.07% received inappropriate shocks. Some of these inappropriate shocks from ICDs implanted for Brugada syndrome are due to supraventicular arrhythmias [2]. In the study by Veltmann [14], 0.4% per year received inappropriate shocks due to atrial fibrillation. Measures to prevent inappropriate ICD shocks due to AF are dual chamber ICDs and rate lowering drugs [9]. This is especially important in view of the findings of the DATAS trial in which dual chamber ICDs reduced clinical adverse events related to atrial fibrillation when compared with single chamber ICDs [15]. In fact, Morita et al [5] have commented that fifth generation ICDs are preferable not only for Brugada syndrome patients with AF but also for Brugada syndrome patients without AF as AF could occur later as AF is more likely to occur in these patients, as already discussed, due to atrial vulnerability. A recent study has shown that quinidine and bepridil are effective in preventing AF in Brugada syndrome [4].

In a very recent study by Pappone et al [16], in patients with new onset atrial fibrillation, 3.2% had Brugada ECG pattern when challenged with flecainide. Of these, one-third had Brugada syndrome. Thus, in new onset lone AF, latent Brugada syndrome may be present.

In short, AF occurs in about 10 to 50% of Brugada syndrome cases. It is more common in those with type 1 ECG pattern and is more common at night. Brugada syndrome patients with AF are more likely to develop syncope and ventricular fibrillation and thus form a high risk subset. Electrophysiological features such as prolonged HV interval and prolonged interatrial conduction time may predict AF in Brugada syndrome. Genetic analysis is not useful for predicting AF. The mechanism of AF in Brugada syndrome may be increased Ito current. Atrial fibrillation is more likely to occur in Brugada syndrome patients with ICD and may cause inappropriate ICD discharges; so dual chamber ICDs are preferable in Brugada syndrome. Brugada syndrome may present as lone AF.

## Long QT syndrome and atrial fibrillation

Ten types of long QT syndromes have been described (LQTS 1 - 10). Long QT syndrome was associated with atrial fibrillation in 1.7% cases in a study done by Johnson [17] in LQTS patients aged < 50 years. This was significantly higher than the 0.1% prevalence of AF in the age-matched general population. The prevalence of AF in LQTS was found to be more in males (3.4%) compared to females (0.7%). The relative risk of developing AF in LQTS compared to the general population was 17.5 in the study. The study probably underestimated the true prevalence of AF in LQTS due to underreporting. Thus probably the prevalence of AF in LQTS is more than reported in this study. Zellerhoff [18] has found that one-third of LQTS patients develop self terminating atrial arrhythmias under daily-life conditions.

In the study by Johnson [17], out of 8 LQTS patients with AF, 5 patients had LQT1, 2 had LQT3 and 1 had LQT7. AF was reported in 2.4% of LQT1 patients (5/211). None of the 174 LQT2 patients had AF. Benito [19] has reported LQT3 with atrial fibrillation in three patients in a family. In two of these patients, AF could be terminated with flecainide. LQT4 is a less common type of LQTS. Mohler  [20] found that it is associated with atrial fibrillation in less than 0.5% cases.

It has been proven that shortening of atrial action potential duration predisposes to atrial fibrillation. But in LQTS, Kirchhof [21] found that atrial action potential duration is increased. The mechanism by which this leads to AF is not clear. It is thought that increased atrial action potential duration leads to atrial polymorphic tachycardia ("atrial torsades de pointes") which mimics AF in the surface ECG.

In LQTS, drugs prolonging the action potential duration, like sotalol and amiodarone, must be avoided. El Yaman [22] has reported a case in which recurrence of atrial fibrillation in an LQT1 patient with ICD implanted was prevented with mexiletine. Mexiletine suppresses late sodium current and shortens action potential duration.

To summarize, long QT syndrome is associated with AF in about 2% cases. The commonest LQTS associated with AF is LQT1. To suppress AF in LQTS, QT prolonging drugs should not be given. Mexiletine may be useful to prevent AF in LQT1; and flecainide may be useful to suppress AF in LQT3, though due to the fact that flecainide produces minimal increase in QT interval, careful monitoring for ventricular arrhythmias is prudent.

## Short QT syndrome and atrial fibrillation

Short QT syndrome (SQTS) was described in 2000 by Gussak [23]. It is characterized by familial incidence of very short QT interval along with atrial fibrillation or sudden death. Short QT interval was defined as corrected QT interval (QTc) less than 300 ms by Gaita [24], as below 340 ms by Anttonen [25] and as less than or equal to 360 ms for men and 370 ms for women by Viskin [26]. Shortness of QT interval becomes evident only at a heart rate less than 80/mt. Anttonen [25] found that presence of short QT in the general population does not predispose them to sudden death, syncope, ventricular tachyarrhythmias or atrial fibrillation. Similar findings were noted by Gallagher [27]. Thus, presence of short QT alone does not define short QT syndrome. It is a separate syndrome caused by specific mutations and forms a subset of all patients with short QT.

In SQTS, atrial and ventricular refractory periods are low. This predisposes the patient to atrial fibrillation and ventricular fibrillation. The mechanism of the short refractory period is gain of function mutation of K+ channels. Three types of SQTS have been described. The genes mutated are KCNH2 in SQT1, KCNQ1 in SQT2 and KCNJ2 in SQT3.

The incidence of atrial fibrillation (AF) in SQTS is about 70% [28].  In the study by Borggrefe M et al [28], the first episode of symptomatic AF occurred at 41+/- 19 years. In about half of SQTS cases, AF was the cause of the first symptoms.

In young patients with lone AF, SQTS has to be excluded as the mean age of presentation is low and AF is the commonest presenting problem. In fact, Poglajen G et al [29] have found that in patients with lone AF, QT interval is significantly shorter than in controls. In this study, QTc < 400 ms was found to be an independent predictor of AF.

ICD implantation is the recommended therapy for SQTS. Gaita F et al [24] have shown that quinidine prolongs QT interval to normal and increases ventricular effective refractory period. Quinidine may be useful as an adjunct to ICD in SQTS. Hong [30] has found that propafenone can maintain SQTS patients free of atrial fibrillation. Propafenone does not normalize QT interval in SQTS. The role of propafenone in the management of SQTS is not clearly defined, but it may be useful to prevent atrial fibrillation even in patients with ICDs as AF is known to cause inappropriate ICD shocks though not proven to do so in SQTS.

Thus, atrial fibrillation is a part of the short QT syndrome. Short QT interval alone does not predispose to atrial fibrillation. In patients presenting with lone AF, SQTS should be excluded. Propafenone can prevent AF in SQTS.

## Familial atrial fibrillation

Familial atrial fibrillation is a recently defined condition. This is a monogenetic disorder in which atrial fibrillation is transmitted in a Mendelian hereditary pattern [31]. It is of 7 types (Table 3) [32-40]. Of these 7 types, ATFB3, ATFB4 and ATFB7 are channelopathies.

ATFB3 is due to gain of function mutation of KCNQ1 gene which codes for alpha subunit of I_Ks_ potassium channel. This leads to increase in I_Ks_, the slow repolarizing potassium current. This leads to decrease in atrial action potential duration and atrial refractory period. This facilitates multiple reentrant circuits leading to atrial fibrillation [41,42].

ATFB4 is due to gain of function mutation of KCNE2 gene which codes for MiRP1. This leads to increase in I_Ks_ leading to atrial fibrillation in the same way as described for ATFB3 [41,42].

ATFB7 is due to mutation of KCNA5 gene which codes for Kv1.5. This leads to loss of IKur, the ultrarapid repolarising potassium current which is found mainly in the atria. This increases atrial action potential duration  and produces early afterdepolarisations in atrial myocytes triggering 'atrial torsades' leading to atrial fibrillation [39,42].

## Channelopathies as the cause of "common" atrial fibrillation

Usually atrial fibrillation occurs outside the setting of the above discussed channelopathies. It occurs due to many causes like valvular heart disease, hypertension and coronary artery disease. Recent research has found that even in these cases, there is a genetic predisposition for AF so that the non-genetic insult acts as a second hit. This may be the reason why only some patients with the above diseases develop AF. Many genetic defects have been shown to predispose to atrial fibrillation in this setting [31]. Of these those affecting channels are:

- KCNE1 (minK) polymorphism - This has been shown to predispose to atrial fibrillation [43,44]. This leads to decrease in I_Ks_. How this leads to AF is not yet clear.
- C825T polymorphism in G protein beta 3 subunit - This predisposes to atrial fibrillation [45] by affecting atrial inward rectifier potassium current.
- SCN5A H558R polymorphism - This has been associated with atrial fibrillation [46]. This leads to decreased sodium channel current leading to shorter wavelength of conducted impulses  [31] which predisposes to atrial fibrillation.

## Conclusions

Atrial fibrillation is an important association of many channelopathies. It has important prognostic and therapeutic implications in these channelopathies. Now it is being increasingly appreciated that even in atrial fibrillation occurring secondary to other diseases, a predisposing channelopathy may be present.

## Figures and Tables

**Table 1:**
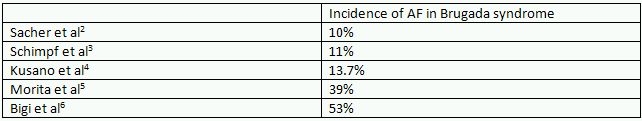
Incidence of AF in Brugada syndrome

**Table 2:**
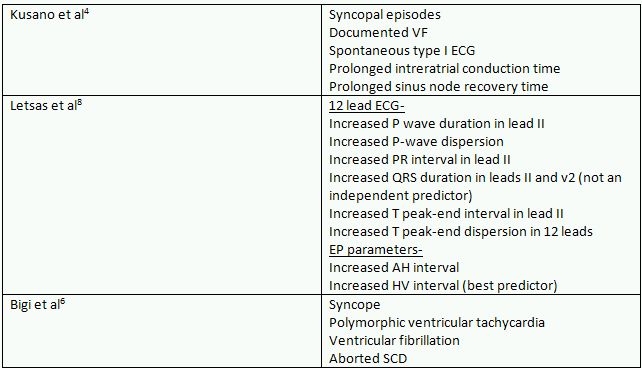
Predictors of atrial fibrillation in Brugada syndrome

**Table 3:**
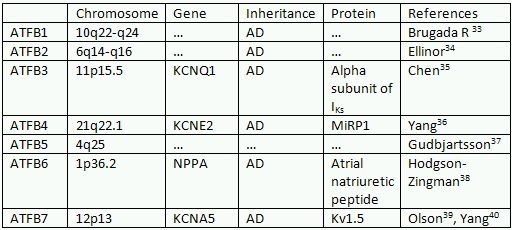
Familial atrial fibrillation
